# Impact of Interventions From a Secondary Home-Based Palliative Care Unit on Caregiver Burden in Kerala, India

**DOI:** 10.7759/cureus.82481

**Published:** 2025-04-18

**Authors:** Manju S Nair, Anupama Augustine, Gopika Nair

**Affiliations:** 1 Centre for Agroecology and Public Health, University of Kerala, Thiruvananthapuram, IND

**Keywords:** caregiver burden, caregiver burden scale, caregiving resources, caregiving services, home-based palliative unit, informal caregivers, palliative and supportive care, palliative medicine research, perceived caregiver burden, secondary palliative care

## Abstract

Introduction: Caregivers in low- and middle-income countries (LMICs) experience severe caregiver burden manifested as physical, emotional, developmental, social, and financial strain. The burden experienced by caregivers becomes severe in the case of patients in need of secondary palliative care since the care recipients are completely dependent on caregivers for activities of daily living (ADLs) and instrumental activities of daily living (iADLs) and require advanced nursing care and symptom management. The paper examines the interventions implemented by the best-performing secondary home-based palliative care (HBPC) unit in Kerala, focusing on the provision of caregiving services and resources, and evaluates their impact on perceived caregiver burden.

Methods: Using in-depth interviews, qualitative data on aspects of caregiver burden and the impact of intervention by the HBPC unit were collected from 10 caregivers selected purposively to reflect the heterogeneity of the caregiver population. A multidimensional tool, the Caregiver Burden Scale (CBS), was developed to measure the impact of the HBPC unit on caregiver burden. A sample of 36 caregivers was selected using proportional random sampling from among the caregivers of 93 patients registered at the HBPC unit in 2021. Quantitative data on palliative care services provided by the secondary palliative care unit, activities of the HBPC unit, frequency and duration of home care visits, particulars of caregivers, and details of caregiving services and resources were collected and analyzed. Additionally, the device CBS was also administered to the selected sample of caregivers.

Results: The domains of caregiver burden impacted by the HBPC unit are found to be time burden, emotional burden, physical burden, social burden, developmental burden, coping up burden, and quality care burden. Of all these domains, notable impact is seen in the developmental and quality care domains. This impact resulted from the HBPC unit targeting the health of caregivers, supporting caregiving services, and providing caregiving resources. The HBPC unit cared for caregivers' physical and emotional well-being and enabled them to maintain social relationships. In addition, medication management, advanced nursing care, and support for ADLs were provided during home care visits. The team, by coordinating with the local self-government (LSG) and other institutions, provided caregiving resources, including care facilities and caregiving education.

Conclusion: The experience of the intervention of the best-performing secondary home-based palliative care team in Kerala depicts that a holistic intervention in the form of provision of caregiving services and caregiving resources from the palliative team can have a positive impact on the various domains of caregiver burden.

## Introduction

Palliative patients suffering from life-limiting illnesses are taken care of mostly at home by family/close relatives in low- and middle-income countries (LMICs). Along with cultural factors, the lack of access to care facilities such as hospices contributes to this practice, creating additional responsibilities for caregivers [1-3]. Caregiver burden, manifested as physical, emotional, developmental, social, and financial strain, is severe in LMICs, where caregivers are solely responsible for managing basic activities of daily living (ADLs) and instrumental activities of daily living (iADLs) for dependent patients [4]. Many caregivers possess minimal knowledge of disease-specific health conditions and have limited access to caregiving resources, including formal training and education [5]. Furthermore, caregiving is often seen predominantly as the responsibility of women [6], who have to balance caregiving duties and household responsibilities.

The magnitude of caregiver burden varies depending on caregiver-specific characteristics, the nature and duration of caregiving services, and access to caregiving resources [7,8]. Caregivers of home-bound palliative patients who are in need of advanced care experience more burden, as these patients are highly dependent on them for ADLs, iADLs, and comprehensive symptom and pain management, often necessitating frequent medical attention. This results in severe physical, emotional, developmental, social, and financial strain on caregivers [9]. Government and community support in providing for complex symptom management, specialized nursing care, and advanced palliative care, based on guidelines or policies, could alleviate this burden [10]. Against this backdrop, the need for a specialized palliative care team delivering holistic care at home becomes increasingly significant.

Kerala, a pioneer state in popularizing the primary care approach to palliative care, has incorporated home-based palliative care (HBPC) units as part of its efforts to expand palliative care coverage. The Pain and Palliative Care Policy of the Government of Kerala (GoK) 2019 incorporates the provision of effective home-based palliative care services to everyone in need, regardless of their demographic, social, economic, and financial status [11]. As such, a decentralized palliative care structure is designed where the provision of palliative care is attached to primary, secondary, and tertiary public health facilities. The HBPC unit is formed and attached to palliative units in primary and secondary public health facilities, under the support and monitoring of the respective local self-government (LSG) [12]. HBPC units attached to the primary palliative units focus on medication management and nursing care, while those attached to secondary palliative units provide advanced nursing care and physiotherapy services [11].

The HBPC unit attached to secondary-level public health institutions, namely, community health centers (CHCs), envisions patient-centered, advanced palliative care provision at home. The home care team includes a doctor, palliative nurse, physiotherapist, and Accredited Social Health Activists (ASHA) and conducts monthly home care visits. During homecare visits, the palliative nurse from the primary palliative unit, elected members from the respective LSG, and volunteers accompany the group. This multisectoral team develops a holistic palliative care plan for patients in consultation with caregivers. This includes pain and symptom management, medication management, physiotherapy services, and advanced nursing care.

Although the HBPC unit focuses primarily on the patients, they can also help alleviate caregiver burden by addressing the caregiver's health, offering caregiving support services, and providing caregiving resources [13-15]. Interaction with the home care team can reduce psychological distress and social isolation experienced by the caregivers, while regular health checkups can enhance their physical health [16]. Similarly, guidance provided to caregivers on medication management, ADLs, and nursing care can enhance the overall experience of both patients and caregivers.

Studies relating to the impact of HBPC units primarily focus on the quality of life of palliative patients, often overlooking the effect on caregivers, despite a strong correlation between the two [17]. Research on caregiver burden shows mixed results; some findings indicate a positive impact on reducing caregiver burden, particularly in the psychosocial aspects, by enhancing the quality of caregiving services and providing a supportive environment through listening and kindness [18,19]. However, other studies indicate that intervention from a multisectoral palliative home care team does not necessarily reduce caregiver burden [20], and most often, caregiver needs remain unaddressed during home care visits [21]. Against this background, this paper examines the interventions done by the best-performing secondary HBPC unit in Kerala, focusing on aspects of caregiving services and resources provided as well as the consequent impact on caregiver burden.

## Materials and methods

The HBPC unit of CHC at Vilappil, recognized as the best-performing secondary palliative care unit in the state, has been identified for a comprehensive assessment of the impact of secondary HBPC units on caregiver burden. The HBPC unit was purposively chosen to highlight the practices initiated by the unit to target caregiver burden, which other palliative units can adopt. Although the secondary palliative care unit was established in 2019, it became fully operational only in 2021, as the COVID-19 pandemic affected its functioning.

A total of 93 patients were registered during the year 2021 with the distribution of disease patterns of cancer (13.16%), chronic kidney disease (23.68%), stroke (34.21%), paraplegia (21.05%), quadriplegia (2.63%), prostate enlargement (2.63%), and others (2.63%). Of the patients, 40% (38 patients) registered in 2021 were selected using the proportional random sampling method to reflect the disease pattern. Of the 38 selected patients, quantitative data were collected from 36 caregivers, as two patients each had a single caregiver. This included a woman taking care of her husband in a paraplegic condition and her son suffering from cancer, and another caregiver caring for her mother, a cancer patient, and her husband suffering from chronic kidney disease. The data collection period was from August 2023 to January 2024. The sample selection criteria ensured that the palliative patients received continuous support from the secondary palliative unit for at least two years at the time of data collection. Data on palliative care services provided by the CHC, activities of the HBPC unit, frequency and duration of home care visits, particulars of caregivers (including demographic, socioeconomic, and disease profiles), and details of caregiving services and resources were collected from the caregivers.

To understand the dimensions of the interventions of the HBPC unit on caregiver burden, in-depth interviews were conducted with 10 caregivers, selected using an inclusion criterion. The selection considered both patient- and caregiver-specific characteristics, and inclusion criteria comprised caregivers caring for patients with low, medium, and high dependence categories on ADLs (two caregivers each from three categories) and one caregiver each from different age categories (below 40, 40-60, 61-75, and above 76), to have a good representation of the characteristics of the caregiver population.

Interviews focused on unearthing information relating to the nature of caregiver burden, nuances of home care visits and the rapport with the home care team, and caregiving services and resources provided by the team and their impact on caregiver burden. Information gathered from the interviews was coded, and resultant themes were identified. A multidimensional tool, the Caregiver Burden Scale (CBS), consisting of 38 items across seven domains, was developed to measure the perceived caregiver burden after the intervention from the HBPC unit, based on identified themes. The scale for each item on CBS ranged from one to five, with one depicting a strong negative impact, three depicting no impact, and five depicting a strong positive impact due to the intervention from the HBPC unit. The CBS was administered to 36 caregivers, and the results were evaluated for reliability using Cronbach's alpha. Informed consent was obtained from the caregivers before collecting data, and the study was approved by the University Level Ethics Committee (approval number: EC/NEW/INST/2022/2586).

## Results

Caregiver-specific characteristics

Caregiver-specific characteristics, such as gender, age, and educational and occupational status, are summarized in Table 1. The demographic profile of caregivers indicates that 80% of the caregivers are female. Around half of the caregivers are middle-aged, and 36% belong to the elderly category. Two caregivers belong to the very old category, burdened with caregiving responsibilities at an age when they themselves need care. The relationship status of caregivers shows that half of them are wives. Thus, most caregivers are married females with low educational attainment who shoulder both household and caregiving responsibilities.

**Table 1:** Socioeconomic and demographic profiles of caregivers

Sociodemographic characteristics	Number (%)
Gender	
Male	7 (19.44)
Female	29 (80.55)
Education	
Primary/upper primary	27 (75.00)
Higher secondary	4 (11.11)
Graduation	5 (13.89)
Age	
Below 40 years	6 (16.67)
40-60 years	17 (47.22)
61-75 years	11 (30.56)
76-90 years	2 (5.56)
Relationship status	
Daughter	3 (8.33)
Daughter-in-law	2 (5.56)
Husband	7 (19.44)
Mother	3 (8.33)
Mother-in-law	1 (2.78)
Sister	2 (5.56)
Wife	18 (50.00)
Economic status	
Above poverty line	7 (19.44)
Below poverty line	29 (80.56)

The employment status of caregivers (Table 2) shows that the percentage of unemployed caregivers has increased to 50%, compared to 16.67% as a result of taking up caregiving responsibilities. Of the employed caregivers, the majority are in wage employment, and one caregiver has given up her semigovernmental job to assume caregiving responsibilities. Interaction with caregivers revealed that they have moved from skilled to non-skilled jobs due to difficulties in fulfilling their job responsibilities. Most caregivers are employed in the Mahatma Gandhi National Rural Employment Guarantee Programme (MNREP), an employment guarantee program implemented by the central government. As the care receivers are highly dependent on the caregivers for ADLs, pain control, and symptom management, many caregivers quit their jobs to perform caregiving responsibilities even amid financial hardships.

**Table 2:** Previous and current employment status of caregivers

Category	Previous employment (number (%))	Current employment (number (%))
Self-employment	1 (2.78)	1 (2.78)
Wage employment	16 (44.44)	14 (38.88)
Semigovernmental	3 (8.33)	1 (2.78)
Private	10 (27.78)	2 (5.56)
Unemployed	6 (16.67)	18 (50.00)
Total	36 (100.00)	36 (100.00)

Intensity of caregiving and caregiver burden

The intensity of caregiving is dependent on the time spent on caregiving activities, the support provided for performing ADLs and iADLs, and the burden experienced by the caregiver while performing these activities [22]. The disease details of patients given in Table 3 show that 70% of the patients are suffering from life-limiting/prolonged illnesses such as cancer, chronic kidney disease, and stroke, which need a cautious symptom management plan, advanced nursing care, and physiotherapy services, along with good medication and doctor consultation. Around 24% of patients are experiencing paraplegia and quadriplegia, showing that they are completely dependent on their caregivers for performing ADLs and iADLs, and caregivers have to be with these patients throughout the day. Caregivers are also responsible for taking patients to recurring hospital visits for consultations/inpatient care and advanced nursing care. The time and effort for recurrent hospital visits, costs incurred for caregiving resources, doctor consultations, medication, and healthy diet increase caregiver burden. Additionally, these expenses create financial hardships for caregivers as they have to manage palliative care costs and household expenses simultaneously.

**Table 3:** Disease details of patients

Disease category	Number (%)
Cancer	5 (13.16)
Chronic kidney disease	9 (23.68)
Stroke	13 (34.21)
Paraplegia	8 (21.05)
Quadriplegia	1 (2.63)
Prostate enlargement	1 (2.63)
Other	1 (2.63)
Total	38 (100.00)

Caregivers of multiple care receivers experience a severe burden managing these chronic illnesses simultaneously. When both care receivers are equally vulnerable and are dependent on daily living on the caregiver, the time, energy, and money for fulfilling the responsibilities are doubled. Also, the caregiver finds it difficult to manage the conflicting interests of both patients and prioritize the caregiving responsibilities of each patient.

Caregiver Burden: Physical, Emotional, Developmental, Social, and Financial Functioning

The physical health problems suffered by caregivers worsen caregiver burden. The disease details of the caregivers are given in Table 4. Of the total caregivers, around 70% suffer from diseases, with high blood pressure and diabetes being the prevalent diseases. Around 25% of the caregivers suffer from chronic illnesses such as cancer, cardiac issues, coronary artery disease, and arthritis.

**Table 4:** Disease details of caregivers Source: Primary data

Disease category	Number (%)
Coronary artery disease	2 (5.56)
Cardiac issues	2 (5.56)
High blood pressure	9 (25.00)
Diabetes	7 (19.44)
Arthritis	3 (8.33)
Cancer	3 (8.33)
None	10 (27.77)
Total	36 (100.00)

The experiences of very old caregivers being entrusted with caregiving responsibility in the absence of a healthy caregiver deepen the caregiver burden.

“Our only daughter drowned to death at a very early age, and we both live here alone. He was diagnosed with rectum cancer 10 years back, and now, I am getting old. We don’t have anybody to look after us, so we depend on government hospitals for treatment and public transport for travel. We can’t do any strenuous job, so we are selling water lilies to earn a living, which provides a very meagre income.” - Caregiver 01

In addition to the physical health problems, the mental strain experienced by caregivers is the most severe, especially when more than one member of the family suffers from chronic disease and the caregivers have to care for multiple patients.

“I am 60, and now, I have to take care of both my husband and son. My husband has been suffering from paraplegia for the last 20 years, and my son is suffering from cancer. I am unable to cater to the needs of both, and this leads to mental stress and restlessness.” - Caregiver 02

While performing their responsibilities, most caregivers go through a heavy schedule of work that starts in the early morning and lasts till night. This busy schedule of caregiving responsibilities, along with household chores, leaves them isolated. They are unable to meet friends or relatives and cannot attend any function or take some time off. This leads to strained relationships within and outside the family. The social isolation, in turn, increases psychological distress and hopelessness in life. Many caregivers experience significant social isolation, feeling too shy to ask for help from the community for necessities such as food or medicine. This, in turn, affects the developmental domain of caregivers, where they have to sacrifice their life goals to fulfil their caregiving responsibilities. This becomes evident in the case of caregivers who are young and uncertain about the future course of life.

“I have completed my degree and wanted to continue my studies, but after my mother became paralyzed, I am not able to do so. Now, I am confused about how to proceed with life.” - Caregiver 09

Medical expenses incurred for prolonged periods can put families into a debt trap. Most patients registered in the palliative unit were once the breadwinners of their families, and once they became bedridden, the paucity of financial resources affected the education, healthcare, and food expenses of other family members, resulting in borrowing/distress financing, which led to a poverty trap. Some caregivers are forced to take on paid work in addition to caregiving and household responsibilities and are severely burdened. Of the total households, 74% suffer from financial liabilities.

“We had to do multiple surgeries, and the treatment cost was very high. Initially, we borrowed from some friends and relatives and later had to pledge our home. He (the care receiver) was the only one with a job, and now, we are finding it difficult to meet the day-to-day expenses. The debt amount is huge, and we are unable to pay even the interest, and at times, they (lenders) turn bitter and blame us.” - Caregiver 02

Impact on caregiver burden: intervention from the secondary home-based palliative care unit

The HBPC unit attached to the palliative unit, CHC, Vilappil, caters to the needs of patients by ensuring monthly home care visits for all registered patients. The patients are referred from primary palliative units and require advanced nursing care and physiotherapy services, and the team provides these services at home. The home care team ensures the doctor's consultation whenever necessary and guarantees inpatient care and physiotherapy in the palliative unit, attached to CHC. This helps reduce the number of recurrent hospital visits and the cost incurred, as these patients previously had to visit a hospital to access these services. The HBPC unit attached to the palliative unit is run from the budgetary provision earmarked for palliative care in the annual budget of the second-tier LSG, along with financial support from the National Health Mission (NHM), a centrally sponsored scheme [23]. The functioning of the unit is monitored by committees such as the Palliative Management Committee and the Palliative Care Committee, which include both members from LSG and secondary care facilities as well as palliative care units [24]. This makes the functioning of the HBPC unit well-planned and coordinated, with the services being provided consistently and with quality.

The monthly house visits conducted by the HBPC unit provide advanced nursing care, including difficult catheterization, tracheostomy care, colostomy care, stroke care, and medicines. The team ensures that doctors' consultations are available and provides assistive devices and care resources according to each person's needs. The nuances of the intervention of the HBPC unit and the perceived impact as reported by caregivers are detailed in Table 5.

**Table 5:** Intervention of HBPC and impact on caregiver burden: identification of themes and domains HBPC: home-based palliative care, MGNREGA: Mahatma Gandhi National Rural Employment Guarantee Act 2005, CSOs: civil society organizations

Quotes	Themes	Domains
Caregiver 01: “They (HBPC team) change the diapers, replace urine and colostomy bag, give medicines and check the vitals, and show us how to care/replace these devices, … so that now, I can plan accordingly to change these tubes, and the time taken for these activities has come down.” Caregiver 05: “Earlier, I had to take (care receiver) to the hospital for physiotherapy, and it was physically and financially burdensome. Now, the team provides physiotherapy at home free of cost, and it’s a great relief, and I find time for managing my activities and household work.”	Time for self-care and household work	Managing time burden
Caregiver 04: “She (palliative nurse) is very friendly. We like her smile. Our friends and family visit us, but her visit is something special; the only person we happily wait for is her.” Caregiver 02: “When the team visits us, I share all my tensions, pain, and family issues with them. I am worried about the rift within the family about how to deal with my son’s cancer. Upon my request, she (the nurse) talked to my family members and tried to convince them.”	Feeling of happiness, caring, and reduced anxiety/sadness	Managing emotional burden
Caregiver 05: “They are also concerned about my health and asked me to check the cholesterol and sugar level frequently and take medications as needed.” Caregiver 06: “She (nurse) checks his (patient’s) wounds, and last time, there was swelling in the legs. Then she took a picture of it and sent it to the doctor and convinced the doctor to visit us. This helps to reduce the difficulty of recurrent hospital visits.” Caregiver 08: “I have to look after my aged mother, husband, and the younger son, along with being the caregiver for my elder son. I get up early to cook and do household chores, and then go for MGNREGA work. When the nurse comes, seeing my tiredness, she monitors my vitals and insists I have food on time.”	Improvement in the health of caregivers due to decreased responsibilities	Managing physical burden
Caregiver 02: “Talking with the nurse makes me happy and confident. It gives a lot of willpower. We ask her opinion about the treatment options and medications, and her guidance helps us to get out of this redundancy of life.” Caregiver 07: “She is the only one who supports my aspirations, recognizes my abilities, and helps me to dream a better life.” Caregiver 07: “I don’t like to mingle with neighbors and family members; I don’t trust them. The team was the first one who supported us when we were in a helpless condition. When they first came here, we both (caregiver and care receiver) were in a pathetic condition. We were surviving on tap water; we had nothing to eat and no fuel to cook. The physiotherapist cared for my mother, dressed my mother, and gave us food, and the nurse gave me first aid when I fainted due to ill health and offered to help me to see a doctor and do an ultrasound scan for the health issues.”	Decision-making and managing life goals	Managing developmental burden
Caregiver 03: “Finding no one to support us, they talked to nearby CSOs and raised some funds to help us. The only social event we attend is the family get-together program for palliative patients, and we cherish talking with other patients and feel like we are a part of the community.”	Socialization of caregiver	Managing social burden
Caregiver 02: “They ask us to be positive and to take care of our health as much as we can.” Caregiver 07: “I was not able to accept things (paraplegia of mother) that happened in my life, and conversations with the nurse helped me to think maturely and to understand my responsibilities as a caregiver.”	Managing life goals and responsibilities	Coping up burden
Caregiver 10: “Initially, we never liked anyone coming to our home, seeing our condition. We used to be annoyed. But they never gave up; they patiently talked to us and started physiotherapy. Now, there is an improvement in our daily routine, diet pattern, medications, and daily activities. Everything has changed. Now, I can communicate with my care receiver positively, and she is also happy.”	Caregiving activities and enhanced quality	Quality burden

The excerpts of the caregiver interview reveal the aspects where the HBPC unit intervened and show the relationship between the care team and the caregivers. During the house visits, in addition to providing physiotherapy services and nursing care, the team made special efforts to educate the caregivers so that they could become skilled in providing caregiving services. Additionally, the team was able to build rapport with the household members that they began to trust the team and shared their concerns. This, in turn, helped in developing a comprehensive care plan for the patient and provided emotional support for the caregivers. The caregiver's health was also monitored by the team, and they were advised to maintain their physical health. Another aspect the home care team targeted is addressing developmental burden; caregivers feel desperate not being able to fulfil their dreams or make informed decisions related to caregiving responsibilities or life goals. The team was prudent enough to inspire the caregivers in such moments and provided them with resources and other facilities, making them optimistic. Other than the medical support, arenas for social gathering and financial support were also offered by the team. Intervention from the team has helped caregivers to improve their ability to cope with the circumstances and enhance the quality of caregiving.

As mentioned in the methodology, the identified themes and domains were used to construct the CBS, and the tool was administered to 36 caregivers. The results are depicted in Table 6. A mean score of more than three shows a positive impact of the HBPC unit on the domains. The domains include time burden, emotional burden, physical burden, developmental burden, social burden, coping up burden, and quality burden. Mean scores above three for all items across all scales show that there has been a reduction in caregiver burden because of the intervention of the HBPC unit. Developmental burden and quality burden domains are the two aspects that witnessed significant impact, with a mean score above four. The domains coping up burden, physical burden, and emotional burden were also impacted by the intervention from the HBPC unit. The results confirm the findings from the qualitative data regarding the ability of the HBPC team to influence the burden on caregivers of secondary palliative care patients.

**Table 6:** Results of CBS: mean scores after registering with the HBPC unit CBS: Caregiver Burden Scale, HBPC: home-based palliative care, SD: standard deviation, ADLs: activities of daily living

Item	Mean	SD
Time burden	3.33	0.41
I require less time to complete caregiving activities.	3.63	0.59
I have more time for self-care activities.	3.55	0.55
I have more time for managing household responsibilities.	3.11	0.46
I can find time to manage work responsibilities and social life.	3.02	0.37
Emotional burden	3.70	0.57
My level of happiness has improved.	3.94	0.62
My fear of the future/negative thoughts about life has reduced.	3.66	0.71
My concern over choosing the correct palliative care choices has reduced.	3.80	0.66
The feeling of sadness/depression has reduced.	3.61	0.59
Experiencing an improved sense of relief as the team takes care of the care receiver with love and respect.	3.45	0.61
Physical burden	3.71	0.52
My physical discomfort has reduced.	3.97	0.65
The feeling of fatigue and the burden of responsibilities have reduced.	3.5	0.60
The dependence of my patient on ADLs has reduced.	3.63	0.59
The physical strain of recurrent hospital visits and travel has reduced.	3.72	0.56
I am able to consume more nutritious food.	3.75	0.60
Developmental burden	4.32	0.62
Enhanced decision-making capacity due to awareness of medication and side effects.	4.55	0.77
Better information to tackle episodes of pain and symptom distress.	4.22	0.59
More committed to aspirations and life goals.	4.61	0.76
Calmness to deal with complex issues in family relationships.	4.11	0.70
Improved decision-making skills in adjusting the household responsibilities alongside care responsibilities.	4.13	0.79
Social burden	3.63	0.38
Improvement in social interactions/support networks.	3.88	0.52
Able to participate in family functions and socialize more.	3.80	0.46
More people in the neighborhood/community acknowledge and support us.	3.36	0.54
More support from the church/other palliative care facilities.	3.50	0.65
Coping up burden	3.88	0.44
Ability to keep social relations intact.	3.61	0.64
Capacity to control the grief and upheaval brought on by a particular event.	3.97	0.44
Enhanced ability to approach a problem in a novel and helpful way.	3.75	0.55
To some extent, anger and embarrassment about life have changed into optimism.	4.05	0.47
The feeling of being on edge, jumpy, or easily startled after a conversation with the patient has reduced.	3.91	0.64
The feeling of having all responsibilities on my shoulders has reduced.	3.88	0.52
Ability to think logically on the verge of worsening symptoms for the patient and taking care decisions has improved.	3.97	0.44
Capacity to accept changes in life after the incidence of chronic illness.	3.94	0.47
Quality burden	4.21	0.62
Respecting the dignity and aspirations of the patient.	4.16	0.65
Supporting ADLs of patients and assisting them to gain/enhance daily life skills.	4.16	0.56
Sharing the disease details and palliative care plan with the patient.	4.25	0.69
Answering the queries of patients and listening to them.	4.58	0.76
Spending more quality time with the patients.	3.94	1.21
Appreciating the patients for the small improvements/achievements.	4.25	0.76
Following a diet plan and conducting routine checkups.	4.16	0.84

The reliability of CBS was assessed using Cronbach's alpha, an internal consistency measure. The results for each caregiver burden domain are shown in Table 7, depicting the reliability of the tool.

**Table 7:** Results of the reliability test: Caregiver Burden Scale

Domain	Item	Cronbach's alpha
Time burden	4	0.83
Emotional burden	5	0.93
Physical burden	5	0.91
Developmental burden	5	0.90
Social burden	4	0.63
Coping up ability	8	0.94
Quality of caregiving	7	0.88

## Discussion

Caregiver burden is multifaceted and complex and is affected by caregiver-specific characteristics, the intensity of caregiving services, and access to caregiving resources. Results from the primary data reveal important insights into the aspects of caregiver burden and agree with many of the earlier findings. The caregiver-specific characteristics of the study show that half of the caregivers are women, and they are either middle-aged, old, or very old, in need of support to maintain their own physical/mental health. This aligns with the existing studies depicting women, particularly middle-aged and elderly, being the caregivers for palliative patients [25,26]. Burden is experienced in domains such as time, emotional, physical, developmental, and social functioning [27]. Also, the study agrees with the earlier findings that caregivers mostly experience challenges in managing complex family and social relationships and identifying care programs and welfare programs amid high dependency by care receivers for ADLs [5].

The HBPC unit, Vilappil, targeted all three aspects to alleviate the caregiver burden while caring for patients: monitoring the caregiver's health and well-being, providing caregiving services, and delivering caregiving resources. The team tried to monitor the caregiver's health by assessing physical health, taking care of the psychological distress experienced, and trying to build social relations. Along with this, the team tied up with other agencies to reduce the financial hardships of caregivers and improve living conditions. The team ensured medication, nursing, and physiotherapy services and supported ADL while on house visits. In addition, the HBPC team worked in coordination with all levels of the palliative service delivery system to educate the caregiver and execute a comprehensive palliative care plan. The particulars of the intervention from the HBPC unit are summarized in Table 8.

**Table 8:** Intervention strategies of the HBPC unit HBPC: home-based palliative care, ADLs: activities of daily living, LSG: local self-government

Aspects targeted	Intervention from the HBPC unit
Caregiver's health and well-being	
Physical health	Checkup on demand for caregivers (blood pressure, temperature, etc.), diet plan for staying healthy
Psychological distress	Creating rapport with caregivers and other family members, counselling in need
Social relationships	Palliative care patients get-together program, maintain good contact with patients' neighborhood
Living conditions	Networking with other agencies to provide support for social determinants of health
Time off/break to rest	Spending additional time in need so that the caregivers can relax during home visits
Caregiving services	
Medication	Medication free of cost, including opioids
Support for nursing care	Physiotherapy services, catheterization, lymph edema management, tracheostomy care, etc.
Support for ADLs	Helping the patient with bathing, dressing, and eating
Caregiving resources	
Care facilities	Colostomy bags, wheelchairs, air bed, cerebral palsy chair, walker, ankle-foot orthoses
Nutrition and health needs	Protein powder, toiletries, food kit
Financial resources	Fund from LSG for care resources, associating with civil society organizations, schools, and private companies to raise additional funds
Knowledge of prospective palliative care options	Identification and referrals of beneficiaries from the primary health center to the secondary health center, provision of care for discharged patients from tertiary facilities
Caregiving education	Training and awareness related to cleaning wounds, treating bedsores, changing urine bags, checking vitals, and symptom management
Awareness of insurance and government welfare schemes	Awareness of old age pension, government welfare schemes, health insurance schemes, rehabilitation facilities, and communication with LSG officials on instances of delay, and inclusion in the "extremely poor category" by LSG

The results of the study corroborate the conclusion of earlier studies that intervention from the HBPC unit impacts various domains of caregiver burden, particularly the developmental burden of caregivers [28]. The study also reveals that intervention can impact the coping up burden of caregivers and the quality of caregiving services. In this regard, the direct provision of caregiving services and resources supported by LSGs is a better strategy, as demonstrated by the sample HBPC unit. For instance, an initiative from the LSGs to include registered palliative patients in need of financial help in the category of "extremely poor" to benefit from government welfare schemes is a good example. The study supports earlier findings on the significance of caregiver education and its capacity to reduce the burden by enhancing their skills to provide caregiving services effectively [29,30].

One aspect where the HBPC unit has made a minimal impact is in reducing the time burden of caregivers. Increasing the frequency of home care visits by the HBPC unit can positively impact the time burden. There is no provision for a bystander facility in need from the HBPC unit, which could otherwise offer temporary relief to caregivers. Hence, caregivers have limited mobility, adversely affecting their ability to perform other responsibilities.

Study limitations

The study findings are based on self-reported data from caregivers, which may cause bias in assessing the caregiver burden. Additionally, the long-term impact of the intervention from the HBPC unit is not examined, as the participants have only been receiving support since 2021. The findings are context-specific to the selected HBPC unit and are not fully generalizable to other palliative units in the state.

## Conclusions

In LMICs, palliative patients are often cared for at home due to the absence of hospice facilities and cultural factors, with informal caregivers bearing the responsibility, which leads to caregiver burden. These caregivers experience severe strain related to emotional, physical, developmental, and social functioning while fulfilling their caregiving responsibilities. Caregivers of palliative patients in need of secondary care are more vulnerable in this regard since the care receivers are more dependent on the carers for ADLs and iADLs and also demand advanced pain and symptom management. The experience of the intervention of the best-performing secondary home-based palliative care team in Kerala depicts that a holistic intervention from the palliative team can have a positive impact on the various domains of caregiver burden. Findings show that the intervention from the home care team affected all domains, particularly the developmental and quality domains of caregiver burden. The interventions made by the HBPC team reduced caregiver burden by offering caregiving services and resources at home, along with addressing their health needs. These interventions, in turn, reinforced better emotional, physical, social, and developmental functioning of caregivers and improved their coping ability and quality of caregiving.

## Appendices

Secondary home-based palliative care unit and its impact on caregiver burden

Interview Schedule (Caregiver)

Age of the caregiver:

Gender of the caregiver:

Relationship with the patient:

Educational qualification of the caregiver:

Occupation of the caregiver:

Age of the care receiver:

Disease of the care receiver:

Duration of caregiving:

Palliative Performance Scale (PPS) level of the care receiver:

Whether supporting the care receiver for ADLs:

Specify the caregiving activities:

The Caregiver Burden Scale used in the study is presented in Table 9.

**Table 9:** Caregiver Burden Scale HBPC: home-based palliative care, ADLs: activities of daily living

Number	Item	Rating (strong negative impact: 1, negative impact: 2, no impact: 3, positive impact: 4, strong positive impact: 5)
	Time burden	
1	Did the support and training from the HBPC unit help you complete caregiving activities in less time?	
2	Do you get more time for self-care activities after registering with the HBPC unit?	
3	Are you able to manage household responsibilities more efficiently after registering with the HBPC unit?	
4	Do you feel you can balance work responsibilities and social life better after getting support from the HBPC unit?	
	Emotional burden	
1	Have you experienced more happiness since receiving support from the HBPC unit?	
2	Did the support from the HBPC unit help you alter the fear of the future/negative thoughts of life?	
3	Have you been able to make informed choices about palliative care services since registering with the HBPC unit?	
4	Did the feelings of depression and sadness change after interacting with the HBPC unit team?	
5	Do you feel relieved at the thought that the HBPC unit team is there to take care of the care receiver in any need?	
	Physical burden	
1	After registering with the HBPC unit, did your physical discomfort reduce?	
2	Did your feelings of fatigue and the burden of responsibilities reduce after registering with the HBPC unit?	
3	Do you experience a reduction in the dependence level of the care receiver for ADLs after intervention from the HBPC unit?	
4	Did the physical strain of recurrent hospital visits and travel reduce after homecare visits from the HBPC unit?	
5	Do you feel you consume more nutritious food after receiving support from the HBPC unit?	
	Developmental burden	
1	Do you think your awareness and decision-making capacity have enhanced since the intervention from the HBPC unit?	
2	Do you think you have more access to information relating to pain and symptom distress management after registering with the HBPC unit?	
3	Did the intervention from the HBPC unit inspire you to become more committed to aspirations and life goals?	
4	Has your capability to manage complex family relationships been enhanced since the home care visits of the HBPC unit?	
5	Do you feel that the intervention from the HBPC unit has helped you make decisions that contribute toward balancing household responsibilities alongside care responsibilities?	
	Social burden	
1	Do you think your difficulty connecting with others has reduced since registering with the HBPC unit?	
2	Have you been able to attend family functions or social events more since registering with the HBPC unit?	
3	Do you feel that the HBPC unit intervention has helped to mobilize more support from the neighborhood/community?	
4	Do you think that help from the church and other palliative care organizations has improved since registering with the HBPC unit?	
	Coping up burden	
1	Have you experienced an enhanced ability to keep social relations intact since registering with the HBPC unit?	
2	Do you feel that your control over grief and upheaval brought on by a particular event improved after being registered with the HBPC unit?	
3	Do you believe that after enrolling with the HBPC unit, your ability to approach a problem in a novel and helpful way has improved?	
4	Do you believe that being registered with the HBPC unit has enhanced your capacity to control anger and embarrassment and enhanced optimism?	
5	Do you experience a reduction in the feeling of being on edge, jumpy, or easily startled after a conversation with the patient since registering with the HBPC unit?	
6	Do you think that the feeling of having all responsibilities on your shoulders has reduced after registering with the HBPC unit?	
7	Did the training from the HBPC unit enhance your ability to think logically on the verge of worsening symptoms for the patient?	
8	Do you believe that joining the HBPC unit has helped you be more committed to your aspirations and accept changes in life after the incidence of chronic illness?	
	Quality burden	
1	How have your feelings about providing care and respecting the dignity and aspirations of the patient changed since registering with the HBPC unit?	
2	Do you feel you are effectively supporting the ADLs of patients and assisting them to gain/enhance daily life skills after registering with the HBPC unit?	
3	Do you think that after registering with the HBPC unit, you are able to share the disease details and palliative care plan with the patient?	
4	Is there any improvement in getting on with the person you care for, by answering the queries of patients and listening to them, after registering with the HBPC unit?	
5	Have you been able to spend more quality time with the patient after registering with the HBPC unit?	
6	Do you feel that after receiving support from the HBPC unit, you can appreciate the patient for the small improvements/achievements?	
7	Do you think that your awareness regarding diet plans and conducting routine checkups has improved after registering with the HBPC unit?	

Table 10 outlines the levels of the Palliative Performance Scale (PPS) (Victoria Hospice Palliative Performance Scale version 2 (PPSv2)).

**Table 10:** PPS PPS: Palliative Performance Scale

PPS level	Ambulation	Activity and evidence of disease	Self-care	Intake	Conscious level
100%	Full	Normal activity and work, no evidence of disease	Full	Normal	Full
90%	Full	Normal activity and work, some evidence of disease	Full	Normal	Full
80%	Full	Normal activity with effort, some evidence of disease	Full	Normal or reduced	Full
70%	Reduced	Unable to do normal job/work, significant disease	Full	Normal or reduced	Full
60%	Reduced	Unable to do hobby/house work, significant disease	Occasional assistance necessary	Normal or reduced	Full or confusion
50%	Mainly sit/lie	Unable to do any work, extensive disease	Considerable assistance required	Normal or reduced	Full or confusion
40%	Mainly in bed	Unable to do most activity, extensive disease	Mainly assistance	Normal or reduced	Full or drowsy +/- confusion
30%	Totally bedbound	Unable to do any activity, extensive disease	Total care	Normal or reduced	Full or drowsy +/- confusion
20%	Totally bedbound	Unable to do any activity, extensive disease	Total care	Minimal to sips	Full or drowsy +/- confusion
10%	Totally bedbound	Unable to do any activity, extensive disease	Total care	Mouth care only	Drowsy or coma +/- confusion
0%	Death	-	-	-	-
